# ESKD, Major Cardiovascular Events, and Death Associated With Systemic Inflammation

**DOI:** 10.1016/j.ekir.2026.106364

**Published:** 2026-02-17

**Authors:** Jean-Michel Halimi, Valentin Maisons, Jean-Baptiste de Fréminville, Sébastien Roger, Arnaud Bisson, Stéphanie Chadet, Laurent Fauchier

**Affiliations:** 1 Service de Néphrologie-Hypertension, Dialyse, Transplantation rénale, CHU de Tours, Tours, France; 2 INSERM UMR1327, ISCHEMIA, Université de Tours, Tours, France; 3 INI-CRCT, Tours, France; 4 INSERM UMR1246, SPHERE, Université de Tours, Université de Nantes, Tours, Nantes, France; 5 Service de Cardiologie, CHU de Tours, Tours, France

**Keywords:** arrhythmia, CKD coronary artery disease, epidemiology, systemic inflammation

## Abstract

**Introduction:**

Systemic inflammation may play a role in the progression of chronic kidney disease (CKD) and in the development of major cardiovascular events (MACE). Whether this effect is independent of traditional cardiovascular risks is unclear.

**Methods:**

In this study, we compared the incidence of end-stage kidney disease (ESKD) (i.e., chronic dialysis or renal transplantation), MACE, and death in patients with CKD with high sensitivity C-reactive protein (hsCRP) ≥ 2 mg/l versus hsCRP < 2 mg/l, using the TRINETX platform.

**Results:**

Overall, 163,854 subjects with CKD (defined as estimated glomerular filtration rate [eGFR] < 60 ml/min per 1.73 m^2^, Kidney Disease: Improving Global Outcomes [KDIGO] CKD 3–4) or albumin-to-creatinine ratio > 200 mg/g) (KDIGO A3), hsCRP results, and no concomitant infection were included. After propensity score matching, 27,580 patients with hsCRP ≥ 2 mg/l were compared with 27,580 subjects with hsCRP < 2 mg/l, well-matched for traditional cardiovascular risk factors, including blood pressure, low-density lipoprotein (LDL) cholesterol, baseline treatments, eGFR, and albuminuria. hsCRP ≥ 2 mg/l was associated with a higher risk of MACE (ischemic stroke, myocardial infarction, heart failure, and atrial and ventricular fibrillation) and death (hazard ratio [HR]: 1.941 [95% confidence interval [CI]: 1.853–2.032]) but not a higher risk of ESKD (HR: 1.271 [0.893–1.809]) versus hsCRP < 2 mg/l during follow-up. Among patients with diabetes (21,700 patients), hsCRP ≥ 2 mg/l was associated with higher risks of MACE and death as well as with a higher risk of ESKD (HR: 1.508 [0.997–2.281], *P* = 0.05).

**Conclusion:**

Systemic inflammation is associated with a higher risk of MACE and death among subjects with CKD 3 and 4 or albuminuria (A3), and a higher risk of ESKD among patients with diabetes mellitus, independently of traditional risk factors.

## Graphical abstract

**Figure:**
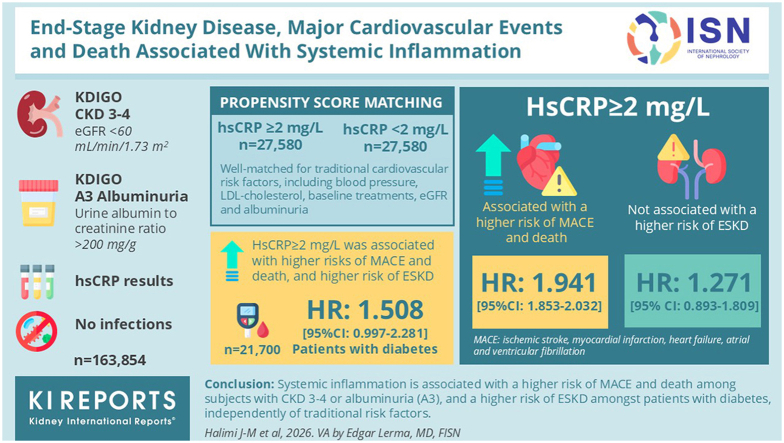

The estimated number of patients with CKD was 860 million subjects in 2017 and will probably increase in the future.^1^ This estimation holds true when CKD is defined as increased albuminuria or mildly reduced eGFR.^2^ In addition to the risk of ESKD, CKD is associated with a high risk of MACE, including coronary heart disease, stroke, peripheral artery disease, arrhythmias, and heart failure.^3^ However, traditional risk biomarkers of atherosclerotic cardiovascular disease are not optimal to predict MACE and death in this population, even after incorporation of glomerular filtration rate and albuminuria in risk prediction scores.^4^

Interestingly, it was recently shown that biomarkers of inflammation were significantly associated with MACE and death among patients with CKD but no cardiovascular disease from the Chronic Renal Insufficiency Cohort, and that this association remained significant after adjustment for baseline renal function.^5^ Moreover, in the CANTOS study, reduction of inflammation using a monoclonal antibody targeting interleukin-1β significantly reduced the risk of recurrent MACE, and this reduction was associated with the magnitude of CRP reduction.^6^^,^^7^ Importantly, this beneficial effect was observed despite no significant change in LDL-cholesterol. In addition, the phase 2 RESCUE trial evaluated ziltivekimab, a monoclonal antibody directed against the interleukin-6 ligand, in patients with systemic inflammation (defined as the elevation of hsCRP > 2 mg/l) and stages CKD 3 and 4.^8^ In these patients, ziltivekimab at 7.5 mg, 15 mg, and 30 mg every 4 weeks over 24 weeks resulted in hsCRP reduction by −77%, −88%, and −92%, respectively versus −4% on placebo. Consequently, a phase 3 trial has been organized in patients with CKD with hsCRP > 2 mg/l to assess the efficacy of ziltivekimab versus placebo on MACE and to prevent progression of renal disease.

However, whether patients with CKD and systemic inflammation have worse renal and cardiovascular outcomes than other patients with CKD, independently of treatment and comorbid conditions is presently unknown. In the present study, we compared the incidence of ESKD, MACE, and death associated with the presence of hsCRP ≥ 2 mg/l (vs. hsCRP < 2 mg/l) in patients with CKD, and specifically in the subgroup of patients with diabetes mellitus.

## Methods

### Patient Selection

The data used in this study came from the TriNetX network. It is a global federated health research network providing access to electronic health records, including diagnoses, procedures, medications, laboratory values, and some genomic information for approximately 151 million deidentified patients across 126 large health care organizations in 17 countries worldwide. This analysis included patients with CKD with a significant risk of renal function degradation, defined by eGFR (Modification of Diet in Renal Disease equation) between 15 and 60 ml/min per 1.73 m^2^ (CKD KDIGO stages 3–4) or urinary albumin-to-creatinine ratio > 200 mg/g (KDIGO A3). Among this selected population, the outcomes of patients with hsCRP ≥ 2 (cohort A) and those with hsCRP < 2 (cohort B) were compared. Patients who had a concomitant infection were excluded from the study.

The analysis process included the following 2 main steps: (i) defining the cohorts through query criteria, and (ii) setting up and running the analysis. Setting up the analysis required definitions for the index event (i.e. hsCRP levels), outcomes criteria, and the time frame. This query was run on the Global Collaborative Network with 128 health care organizations queried and 126 health care organizations responded. A total of 80 providers responded with patients. The index event only included events that occurred up to 20 years ago. The follow-up of patients was up to 6 years after inclusion.

### Collected Data

The baseline parameters collected are presented in Table 1. They included demographics, race, and ethnicity; body mass index; blood pressure; cardiovascular comorbidities; acute kidney injury and CKD; diabetes mellitus; overweight and obesity; and noncardiovascular comorbidities, including among others chronic obstructive pulmonary disease, personal history of nicotine dependence, alcohol abuse, and cancers. Laboratory parameters included cholesterol levels, eGFR using creatinine-based formula (Modification of Diet in Renal Disease), albuminuria, and hemoglobin A1c. Collected medication information included presence of beta blockers, calcium channel blockers, angiotensin-converting enzyme inhibitors, angiotensin-receptor blockers, diuretics, antilipemic agents, medications used in diabetes mellitus, anticoagulants, and antiplatelet agents.

**Table 1:** Baseline characteristics of patients before and after propensity score matching

Characteristics	Before propensity score matching				After propensity score matching			
	hsCRP ≥ 2	hsCRP ≤ 2	*P*-Value	Std diff. (%)	hsCRP ≥ 2	hsCRP ≤ 2	*P*-Value	Std diff. (%)
	(*n* = 104,908)	(*n* = 29,473)			(*n* = 27,580)	(*n* = 27,580)		
Age, yr (%)	73.0 ± 12.0	71.7 ± 11.4	< 0.001	10.6	72.7 ± 11.7	72.0 ± 11.3	< 0.001	6.1
Men, *n* (%)	56,281 (53.6%)	16,012 (54.3%)	0.039	1.4	14,923 (54.1%)	14,972 (54.3%)	0.675	0.4
Systolic BP (mm Hg), mean ± SD	126.8 ± 24.2	130.2 ± 23.4	< 0.001	14.2	128 ± 24	130 ± 23	< 0.001	9.8
Diastolic BP (mm Hg), mean ± SD	69.1 ± 14.6	71.4 ± 13.2	< 0.001	16.6	70.1 ± 14.0	71.4 ± 13.2	< 0.001	9
Body mass index (kg/m^2^), mean ± SD	28.9 ± 6.9	26.8 ± 5.4	< 0.001	33.5	27.3 ± 6.0	27.0 ± 5.5	< 0.001	6.2
White, *n* (%)	44,158 (42.1%)	10,646 (36.1%)	< 0.001	12.3	10,430 (37.8%)	10,609 (38.5%)	0.117	1.3
Black or African American, *n* (%)	7409 (7.1%)	1184 (4%)	< 0.001	13.3	1182 (4.3%)	1183 (4.3%)	0.983	0
Asian, *n* (%)	5439 (5.2%)	5634 (19.1%)	< 0.001	43.6	4114 (14.9%)	3821 (13.9%)	< 0.001	3
Hispanic or Latino, *n* (%)	3221 (3.1%)	486 (1.6%)	< 0.001	9.4	485 (1.8%)	484 (1.8%)	0.974	0
Unknown race, *n* (%)	45,488 (43.4%)	11,370 (38.6%)	< 0.001	9.7	11,218 (40.7%)	11,336 (41.1%)	0.307	0.9
Comorbid conditions								
Hypertension, *n* (%)	60,503 (57.7%)	16,833 (57.1%)	0.086	1.1	15,635 (56.7%)	15,661 (56.8%)	0.823	0.2
Diabetes mellitus, *n* (%)	34,126 (32.5%)	9689 (32.9%)	0.265	0.7	8883 (32.2%)	8889 (32.2%)	0.956	0
Smoker, *n* (%)	10525 (10%)	1798 (6.1%)	< 0.001	14.5	1847 (6.7%)	1786 (6.5%)	0.295	0.9
Overweight or obesity, *n* (%)	14,039 (13.4%)	2415 (8.2%)	< 0.001	16.8	2428 (8.8%)	2398 (8.7%)	0.651	0.4
Dyslipidemia, *n* (%)	44,725 (42.6%)	13,962 (47.4%)	< 0.001	9.5	12,878 (46.7%)	12,769 (46.3%)	0.352	0.8
Alcohol-related diagnoses, *n* (%)	1962 (1.9%)	310 (1.1%)	< 0.001	6.8	295 (1.1%)	307 (1.1%)	0.623	0.4
Heart failure, *n* (%)	26,983 (25.7%)	5700 (19.3%)	< 0.001	15.3	5620 (20.4%)	5542 (20.1%)	0.408	0.7
Coronary artery disease, *n* (%)	64,981 (61.9%)	18,680 (63.4%)	< 0.001	3	17,555 (63.7%)	17,493 (63.4%)	0.583	0.5
Myocardial infarction, *n* (%)	8617 (8.2%)	2603 (8.8%)	0.001	2.2	2415 (8.8%)	2480 (9%)	0.33	0.8
Dilated cardiomyopathy, *n* (%)	1643 (1.6%)	329 (1.1%)	< 0.001	3.9	350 (1.3%)	313 (1.1%)	0.148	1.2
Ischemic stroke, *n* (%)	25,320 (24.1%)	6807 (23.1%)	< 0.001	2.4	6312 (22.9%)	6379 (23.1%)	0.498	0.6
Intracranial hemorrhage, *n* (%)	2045 (1.9%)	614 (2.1%)	0.145	1	602 (2.2%)	575 (2.1%)	0.426	0.7
Atrial fibrillation or flutter, *n* (%)	21,952 (20.9%)	4862 (16.5%)	< 0.001	11.4	4711 (17.1%)	4673 (16.9%)	0.667	0.4
Kidney disease, *n* (%)	29,734 (28.3%)	6248 (21.2%)	< 0.001	16.6	60,42 (21.9%)	5823 (21.1%)	0.023	1.9
Lung disease, *n* (%)	42,446 (40.5%)	9798 (33.2%)	< 0.001	15	9428 (34.2%)	9304 (33.7%)	0.265	0.9
COPD, *n* (%)	10,364 (9.9%)	1926 (6.5%)	< 0.001	12.2	1907 (6.9%)	1856 (6.7%)	0.389	0.7
Sleep apnea syndrome, *n* (%)	9259 (8.8%)	2156 (7.3%)	< 0.001	5.5	2100 (7.6%)	2083 (7.6%)	0.785	0.2
Peripheral vascular disease, *n* (%)	10440 (10%)	1848 (6.3%)	< 0.001	13.5	1834 (6.6%)	1817 (6.6%)	0.771	0.2
Previous cancer, *n* (%)	24,016 (22.9%)	7363 (25%)	< 0.001	4.9	6898 (25%)	6774 (24.6%)	0.221	1
Anemia, *n* (%)	13,485 (12.9%)	2936 (10%)	< 0.001	9.1	2929 (10.6%)	2813 (10.2%)	0.106	1.4
Biology measurements								
Total cholesterol (mg/dl), mean ± SD	160.6 ± 50.2	164.1 ± 47.3	< 0.001	7.2	164.5 ± 48.7	163.6 ± 47.7	0.06	1.9
LDL cholesterol (mg/dl), mean ± SD	89.4 ± 38.5	89.5 ± 37.4	0.846	0.2	91.0 ± 37.4	89.1 ± 37.7	< 0.001	5.2
HDL cholesterol (mg/dl), mean ± SD	47.3 ± 17.0	51.6 ± 17.7	< 0.001	24.4	50.2 ± 17.1	51.4 ± 17.9	< 0.001	7
Triglyceride (mg/dl), mean ± SD	136.9 ± 149.1	131.8 ± 225.7	< 0.001	2.7	133.6 ± 147.1	131.9 ± 227.0	0.392	0.9
Hemoglobin A1c (%), mean ± SD	6.5 ± 1.6	6.2 ± 1.4	< 0.001	20.1	6.3 ± 1.5	6.2 ± 1.4	< 0.001	8.4
Estimated GFR (MDRD, ml/min), mean ± SD	50.0 ± 19.3	53.1 ± 15.7	< 0.001	17.3	51.9 ± 17.9	52.8 ± 15.9	< 0.001	5.3
Albuminuria (mg/g), mean ± SD	293.0 ± 939.1	228.6 ± 900.9	0.034	7	187.7 ± 597.2	231.8 ± 913.8	0.164	5.7
Albuminuria > 200 mg/g, *n* (%)	1055 (1%)	274 (0.9%)	0.244	0.8	255 (0.9%)	267 (1%)	0.598	0.4
hsCRP (mg/L), mean ± SD	14.8 ± 24.0	1.0 ± 7.5	< 0.001	-	12.8 ± 21.2	1.0 ± 7.8	< 0.001	-
Hemoglobin (g/dl), mean ± SD	12.2 ± 2.4	13.0 ± 2.1	< 0.001	37.8	12.6 ± 2.3	13.0 ± 2.1	< 0.001	18.6
Baseline treatments								
Beta blockers, *n* (%)	54,391 (51.8%)	13,079 (44.4%)	< 0.001	15	12,659 (45.9%)	12,579 (45.6%)	0.494	0.6
Calcium channel blockers, *n* (%)	37,339 (35.6%)	10619 (36%)	0.166	0.9	9771 (35.4%)	9615 (34.9%)	0.164	1.2
ACE inhibitors, *n* (%)	30,785 (29.3%)	7298 (24.8%)	< 0.001	10.3	7152 (25.9%)	7090 (25.7%)	0.546	0.5
Angiotensin 2 inhibitors, *n* (%)	26,801 (25.5%)	8654 (29.4%)	< 0.001	8.6	7883 (28.6%)	7688 (27.9%)	0.065	1.6
Digitalis glycosides, *n* (%)	4626 (4.4%)	846 (2.9%)	< 0.001	8.2	1033 (3.7%)	807 (2.9%)	<0.001	4.6
Diuretics, *n* (%)	49,490 (47.2%)	10,846 (36.8%)	< 0.001	21.1	10,659 (38.6%)	10,353 (37.5%)	0.007	2.3
Lipid-lowering drugs, *n* (%)	59,401 (56.6%)	16,773 (56.9%)	0.378	0.6	15,501 (56.2%)	15,487 (56.2%)	0.904	0.1
Glucose-lowering therapy, *n* (%)	38,039 (36.3%)	8663 (29.4%)	< 0.001	14.7	8225 (29.8%)	8064 (29.2%)	0.133	1.3
Insulin, *n* (%)	29,279 (27.9%)	5160 (17.5%)	< 0.001	25	5284 (19.2%)	5091 (18.5%)	0.035	1.8
Noninsulin glucose-lowering therapy, *n* (%)	20,592 (19.6%)	5804 (19.7%)	0.807	0.2	5313 (19.3%)	5218 (18.9%)	0.303	0.9
Metformin, *n* (%)	13,453 (12.8%)	4111 (13.9%)	< 0.001	3.3	3625 (13.1%)	3616 (13.1%)	0.91	0.1
Sulfonylureas, *n* (%)	7491 (7.1%)	2022 (6.9%)	0.098	1.1	1843 (6.7%)	1808 (6.6%)	0.549	0.5
GLP-1 receptor agonists, *n* (%)	1826 (1.7%)	537 (1.8%)	0.347	0.6	487 (1.8%)	513 (1.9%)	0.407	0.7
DPP4 inhibitors, *n* (%)	5383 (5.1%)	1796 (6.1%)	< 0.001	4.2	1554 (5.6%)	1546 (5.6%)	0.882	0.1
SGLT2 inhibitors, *n* (%)	3997 (3.8%)	1208 (4.1%)	0.023	1.5	1118 (4.1%)	1103 (4%)	0.745	0.3
Thiazolidinediones, *n* (%)	1440 (1.4%)	596 (2%)	< 0.001	5	426 (1.5%)	518 (1.9%)	0.003	2.6
Antiplatelet therapy, *n* (%)	57,773 (55.1%)	14,945 (50.7%)	< 0.001	8.7	14,377 (52.1%)	14,187 (51.4%)	0.105	1.4
Anticoagulant, *n* (%)	8238 (7.9%)	1188 (4%)	< 0.001	16.2	1261 (4.6%)	1182 (4.3%)	0.102	1.4

ACE, angiotensin-converting enzyme; BP, blood pressure; COPD, chronic obstructive pulmonary disease; DPP4, dipeptidyl peptidase 4; GFR, glomerular filtration rate; GLP1, glucagon-like peptide-1; HDL, high-density lipoprotein; hsCRP, high sensitivity C-reactive protein; LDL, low-density lipoprotein; MDRD, Modification of Diet in Renal Disease formula; SGLT2, sodium glucose cotransporter-2; Std diff., standard difference.

### Sample Size Calculation and Propensity Score Matching

Considering that this was an observational, retrospective real-world evidence study based on the TriNetX Global Collaborative Network, no *a priori* sample size calculation was performed. All eligible patients meeting the predefined inclusion criteria across participating health care organizations were included to maximize statistical power and external validity.

The propensity scores were estimated using logistic regression incorporating all demographic, clinical, laboratory, and medication variables available in TriNetX (57 covariates, as listed in the Supplementary Material). Matching was performed 1:1 using greedy nearest-neighbor matching without replacement, with a caliper of 0.1 of the SD of the logit of the propensity score, as implemented in the TriNetX platform. Balance was assessed using standardized mean differences (< 10% for all variables after matching). The exact number of patients before and after matching was double-checked and corrected, where necessary (27,580 vs. 27,580 in each arm for the main analysis). Propensity score matching was performed on all collected parameters for the whole population, and separately among patients with diabetes mellitus (Supplementary Material).

### Statistical Analyses and Outcome Measures

Results are presented with mean and SD or median and interquartile range for quantitative parameters and number and percentages for qualitative parameters. Kaplan–Meier survival analyses and log-rank tests to compare the risk of these events during follow-up in patients with hsCRP ≥ 2 vs < 2 mg/l were applied for all time-to-event outcomes, with HRs and 95% CIs derived from univariable Cox proportional hazards models. The outcomes were evaluated from 1 day after the index event to 6 years, in accordance with the TriNetX analysis window. For outcomes with chronic baseline conditions, we indicated whether TriNetX excluded patients with previous events (as listed in the Supplementary Appendix generated by the platform). All these clarifications have been added to the revised Methods and Supplementary Material.

Incidence of ESKD (defined as chronic dialysis or renal transplantation), cardiovascular events, and death was assessed over a maximum follow-up of 6 years. For all analyses, we first studied the whole population, and then studied patients with diabetes mellitus.

## Results

### Baseline Characteristics Before and After Propensity Score Matching in the Whole Population

**Figure 1:**
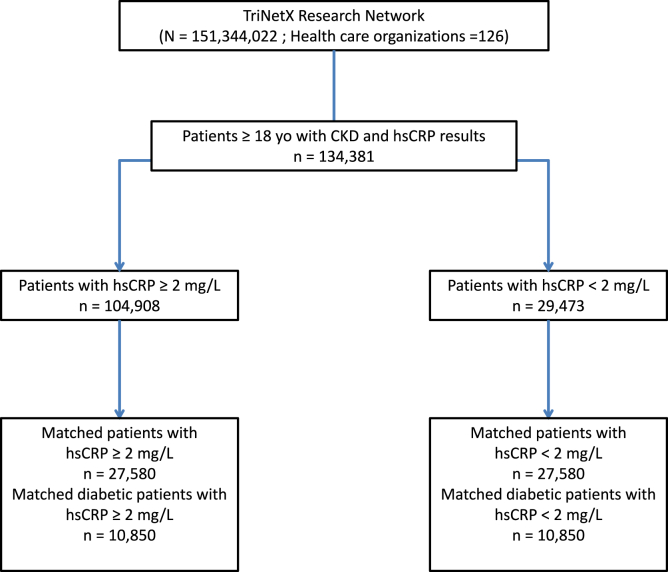
Flow chart. Selected population before and after propensity score matching. CKD, chronic kidney disease; hsCRP, high sensitivity C-reactive protein.

Overall, 134,381 patients had CKD and hsCRP results (104,908 with hsCRP ≥ 2 mg/l and 29,473 with hsCRP < 2 mg/l) (Figure 1). These 2 groups were different for most clinical diagnoses, race, ethnicity, sex ratio, blood pressure, body mass index, comorbid conditions, biological measurements, and treatments (Table 1).

After propensity score matching, 27,580 patients with hsCRP ≥ 2 mg/l were compared with 27,580 patients with hsCRP < 2 mg/l (Table 1, Figure 1). These 2 groups were well-matched (standard difference of all parameters between the 2 groups < 10%) on baseline characteristics (Table 1). In these 2 groups, mean age was 72 years and men represented 54% of the patients (Table 1). No meaningful differences were noted with regard to race and ethnicity, hypertension (57%), diabetes mellitus (32%), body mass index, systolic and diastolic blood pressure, comorbid conditions, smoking habits, and treatments (Table 1). In addition, these 2 populations were well-matched for LDL-cholesterol and high-density lipoprotein cholesterol levels, triglyceride levels, hemoglobin A1c, eGFR, and albuminuria (Table 1). The use of antihypertensive or other cardiovascular medications, glucose-lowering therapy, anticoagulants, and antiplatelet therapy was well-matched (Table 1).

### ESKD, MACE, and Death According to hsCRP Levels During Follow-Up in the Whole Population

The median follow-up was 501 (interquartile range: 1276) days in patients with hsCRP ≥ 2 mg/l and 633 (1428) years in patients with hsCRP ≤ 2 mg/l. As shown in Table 2, patients with hsCRP ≥ 2 mg/l had significant greater risks of all-cause death (yearly rate: 1.94% vs. 10.1%, HR: 1.941 [95% CI: 1.853–2.032]), ischemic strokes, thromboembolism, atrial fibrillation, cardiac arrest, myocardial infarction, and hospitalization for heart failure (Table 2). The rate of ESKD was not statistically different in patients with baseline hsCRP ≥ 2 mg/l versus hsCRP < 2 mg/l (HR: 1.271 [0.893–1.809], *P* = 0.18); however, the risk was low in the 2 groups (yearly rate: 0.21 vs. 0.23%).

**Table 2:** Clinical outcomes during follow-up in the matched population

Outcomes	hsCRP ≥ 2 mg/l		hsCRP < 2 mg/l		Hazard ratio (95% CI)	*P-*value
	(*n* = 27,580)		(*n* = 27,580)			
	Number of events	Yearly rate, %	Number of events	Yearly rate, %		
Death	5012	5.81	2840	3.62	1.941 (1.853–2.032)	< 0.0001
ESKD	67	0.07	57	0.08	1.271 (0.893–1.809)	0.18
MACE and other cardiovascular events						
Ischemic stroke or thromboembolism	173	0.23	118	0.16	1.619 (1.281–2.046)	< 0.0001
Acute MI	142	0.19	101	0.13	1.524 (1.180–1.967)	0.001
Atrial fibrillation	1603	2.55	1217	1.91	1.444 (1.341–1.556)	< 0.0001
VT/VF/cardiac arrest	1147	1.34	839	0.90	1.458 (1.334–1.594)	< 0.0001
Cardiac arrest	521	0.60	224	0.26	2.482 (2.122–2.903)	< 0.0001
VT or VF	707	0.86	668	0.71	1.13 (1.016–1.256)	0.02
MI/stroke/heart failure/death	6617	6.90	4059	4.53	1.797 (1.728–1.869)	< 0.0001
MI, ischemic stroke, or heart failure	2053	1.89	1389	1.26	1.558 (1.455–1.668)	< 0.0001
Incident heart failure	1069	1.95	929	1.61	1.28 (1.173–1.398)	< 0.0001
Hospitalization for heart failure	2063	3.37	1669	2.69	1.355 (1.270–1.445)	< 0.0001

CI, confidence interval; ESKD, end-stage kidney disease; hsCRP, high sensitivity C-reactive protein; MACE, major cardiovascular event; MI, myocardial infarction; VF, ventricular fibrillation; VT, ventricular tachycardia.

### Baseline Characteristics Before and After Propensity Score Matching Among Patients With Diabetes

Overall, 53,078 patients with diabetes had CKD and hsCRP results (41,148 with hsCRP ≥ 2 mg/l and 11,930 with hsCRP < 2 mg/l). Before propensity score matching, most parameters were different between the 2 groups (Table 3). After propensity score matching, 10,850 patients with hsCRP ≥ 2 mg/l were compared with 10,850 patients with hsCRP < 2 mg/l (Table 3). These 2 groups were well-matched (standard difference of all parameters between the 2 groups < 10% for almost all parameters) on baseline characteristics (Table 3). In these 2 groups, mean age was 72 years, and men represented 56% of the patients (Table 3). Patients were similar with regard to race and ethnicity, hypertension, body mass index, systolic and diastolic blood pressure, comorbid conditions, smoking habits, and treatments (Table 3). They were also well-matched for LDL cholesterol and high-density lipoprotein cholesterol levels, triglyceride levels, hemoglobin A1c (6.9% ± 1.6% vs. 6.7 ± 1.6%), eGFR (50.4 ± 18.5 vs. 51.1 ± 16.7 ml/min per 1.73 m^2^), albuminuria (232.8 ± 746.4 vs. 234.7 ± 853.8), and the rate of patients with albuminuria > 200 mg/g (1.9% vs. 2.1%) (Table 3). The use of antihypertensive or other cardiovascular medications, glucose-lowering therapy (including sodium-glucose cotransporter -2 inhibitors and glucagon-like peptide-1 receptor agonists), anticoagulants, and antiplatelet therapy was well-matched (Table 3).

**Table 3:** Baseline characteristics of patients with diabetes before and after propensity score matching

Characteristics	Before propensity score matching				After propensity score matching			
	hsCRP ≥ 2	hsCRP < 2	*P*-Value	Std diff. (%)	hsCRP ≥ 2	hsCRP < 2	*P*-Value	Std diff. (%)
	(*n* = 41,148)	(*n* = 11,930)			(*n* = 10,850)	(*n* = 10,850)		
Age, yr, *n* (%)	71.7 ± 11.6	71.4 ± 10.7	0.01	2.7	72.3 ± 11.1	71.7 ± 10.7	< 0.001	5.6
Men (%)	23,083 (56.1%)	6697 (56.1%)	0.941	0.1	6114 (56.4%)	6080 (56%)	0.642	0.6
Systolic BP (mm Hg), mean ± SD	128.5 ± 24.3	131.4 ± 23.4	< 0.001	12.4	130 ± 24	131 ± 23	< 0.001	7.5
Diastolic BP (mm Hg), mean ± SD	69.0 ± 14.7	71.0 ± 13.0	< 0.001	14.8	70 ± 15	71 ± 13	0.041	4
Body mass index (kg/m^2^), mean ± SD	30.1 ± 7.3	27.5 ± 5.7	< 0.001	40.6	28.1 ± 6.4	27.8 ± 5.9	0.003	5.2
White, *n* (%)	17,045 (41.4%)	3241 (27.2%)	< 0.001	30.4	3127 (28.8%)	3232 (29.8%)	0.117	2.1
Black or African American, *n* (%)	3952 (9.6%)	566 (4.7%)	< 0.001	18.9	586 (5.4%)	566 (5.2%)	0.545	0.8
Asian, *n* (%)	2907 (7.1%)	3016 (25.3%)	< 0.001	51.1	2224 (20.5%)	2069 (19.1%)	0.008	3.6
Hispanic or Latino, *n* (%)	1852 (4.5%)	264 (2.2%)	< 0.001	12.7	268 (2.5%)	264 (2.4%)	0.861	0.2
Unknown Race, *n* (%)	16,137 (39.2%)	4857 (40.7%)	0.003	3.1	4662 (43%)	4735 (43.6%)	0.317	1.4
Comorbid conditions								
Hypertension, *n* (%)	28,675 (69.7%)	7929 (66.5%)	< 0.001	6.9	7236 (66.7%)	7170 (66.1%)	0.343	1.3
Smoker, *n* (%)	4935 (12%)	745 (6.2%)	< 0.001	20.1	748 (6.9%)	740 (6.8%)	0.83	0.3
Overweight or obesity, *n* (%)	8324 (20.2%)	1298 (10.9%)	< 0.001	26	1333 (12.3%)	1290 (11.9%)	0.371	1.2
Dyslipidemia, *n* (%)	23,009 (55.9%)	6722 (56.3%)	0.407	0.9	6057 (55.8%)	6003 (55.3%)	0.461	1
Alcohol-related diagnoses, *n* (%)	797 (1.9%)	100 (0.8%)	< 0.001	9.4	116 (1.1%)	98 (0.9%)	0.216	1.7
Heart failure, *n* (%)	12,895 (31.3%)	3058 (25.6%)	< 0.001	12.7	2911 (26.8%)	2905 (26.8%)	0.927	0.1
Coronary artery disease, *n* (%)	27,658 (67.2%)	7827 (65.6%)	0.001	3.4	7192 (66.3%)	7143 (65.8%)	0.482	1
Myocardial infarction, *n* (%)	3675 (8.9%)	1045 (8.8%)	0.562	0.6	990 (9.1%)	952 (8.8%)	0.366	1.2
Dilated cardiomyopathy, *n* (%)	716 (1.7%)	145 (1.2%)	< 0.001	4.3	131 (1.2%)	138 (1.3%)	0.668	0.6
Ischemic stroke, *n* (%)	9025 (21.9%)	2851 (23.9%)	< 0.001	4.7	2544 (23.4%)	2551 (23.5%)	0.911	0.2
Intracranial hemorrhage, *n* (%)	816 (2%)	311 (2.6%)	< 0.001	4.2	246 (2.3%)	282 (2.6%)	0.113	2.2
Atrial fibrillation or flutter, *n* (%)	8901 (21.6%)	1917 (16.1%)	< 0.001	14.3	1855 (17.1%)	1806 (16.6%)	0.374	1.2
Kidney disease, *n* (%)	15,898 (38.6%)	3381 (28.3%)	< 0.001	21.9	3168 (29.2%)	3087 (28.5%)	0.225	1.6
Lung disease, *n* (%)	18,782 (45.6%)	4383 (36.7%)	< 0.001	18.2	4247 (39.1%)	4093 (37.7%)	0.032	2.9
COPD, *n* (%)	4810 (11.7%)	871 (7.3%)	< 0.001	15	843 (7.8%)	824 (7.6%)	0.628	0.7
Sleep apnea syndrome, *n* (%)	5248 (12.8%)	1073 (9%)	< 0.001	12.1	1028 (9.5%)	1029 (9.5%)	0.982	0
Peripheral vascular disease, *n* (%)	5256 (12.8%)	842 (7.1%)	< 0.001	19.2	838 (7.7%)	824 (7.6%)	0.721	0.5
Previous cancer, *n* (%)	9921 (24.1%)	3365 (28.2%)	< 0.001	9.3	3005 (27.7%)	3004 (27.7%)	0.988	0
Anemia, *n* (%)	6592 (16%)	1402 (11.8%)	< 0.001	12.4	1386 (12.8%)	1320 (12.2%)	0.175	1.8
Biology measurements								
Total cholesterol (mg/dl), mean ± SD	157.9 ± 51.1	163.4 ± 47.3	< 0.001	11.2	163.6 ± 49.7	163.3 ± 48.0	0.704	0.6
LDL cholesterol (mg/dl), mean ± SD	86.4 ± 39.1	88.7 ± 37.2	< 0.001	6	90.2 ± 38.2	88.4 ± 37.6	0.004	4.7
HDL cholesterol (mg/dl), mean ± SD	45.2 ± 16.1	50.4 ± 17.1	< 0.001	31.3	49.0 ± 16.7	50.1 ± 17.3	< 0.001	6.3
Triglyceride (mg/dl), mean ± SD	152.6 ± 160.7	144.6 ± 224.8	0.001	4.1	145.5 ± 120.1	144.9 ± 227.2	0.841	0.3
Hemoglobin A1c (%), mean ± SD	7.2 ± 1.8	6.7 ± 1.5	< 0.001	26	6.9 ± 1.6	6.7 ± 1.6	< 0.001	9.5
Estimated GFR (MDRD, ml/min), mean ± SD	48.3 ± 20.3	51.5 ± 16.5	< 0.001	17.1	50.4 ± 18.5	51.1 ± 16.7	0.002	4.2
Albuminuria (mg/g), mean ± SD	360.0 ± 1081.2	225.5 ± 835.6	0.001	13.9	232.8 ± 746.4	234.7 ± 853.8	0.962	0.2
Albuminuria >200 mg/g, *n* (%)	853 (2.1%)	233 (2%)	0.415	0.9	204 (1.9%)	229 (2.1%)	0.225	1.6
CRP (mg/l), mean ± SD	14.3 ± 21.4	1.0 ± 7.8	< 0.001	82.5	12.4 ± 19.9	1.1 ± 8.1	< 0.001	-
Hemoglobin (g/dl), mean ± SD	12.0 ± 2.4	12.9 ± 2.1	< 0.001	37.4	12.4 ± 2.3	12.8 ± 2.1	< 0.001	18
Baseline treatments								
Beta blockers, *n* (%)	22,774 (55.3%)	5057 (42.4%)	< 0.001	26.1	4853 (44.7%)	4763 (43.9%)	0.219	1.7
Calcium channel blockers, *n* (%)	16,894 (41.1%)	4999 (41.9%)	0.098	1.7	4445 (41%)	4325 (39.9%)	0.097	2.3
ACE inhibitors, *n* (%)	12,667 (30.8%)	2702 (22.6%)	< 0.001	18.5	2596 (23.9%)	2558 (23.6%)	0.544	0.8
Angiotensin 2 inhibitors, *n* (%)	12,661 (30.8%)	4225 (35.4%)	< 0.001	9.9	3669 (33.8%)	3606 (33.2%)	0.365	1.2
Digitalis glycosides, *n* (%)	1817 (4.4%)	353 (3%)	< 0.001	7.7	368 (3.4%)	325 (3%)	0.097	2.3
Diuretics, *n* (%)	20,947 (50.9%)	4533 (38%)	< 0.001	26.2	4349 (40.1%)	4193 (38.6%)	0.03	2.9
Lipid-lowering drugs, *n* (%)	26,139 (63.5%)	7082 (59.4%)	< 0.001	8.6	6375 (58.8%)	6275 (57.8%)	0.169	1.9
Glucose-lowering therapy, *n* (%)	25,703 (62.5%)	6121 (51.3%)	< 0.001	22.7	5660 (52.2%)	5522 (50.9%)	0.061	2.5
Insulin, *n* (%)	18,819 (45.7%)	3159 (26.5%)	< 0.001	40.9	3202 (29.5%)	3100 (28.6%)	0.127	2.1
Noninsulin glucose-lowering therapy, *n* (%)	17,359 (42.2%)	5016 (42%)	0.783	0.3	4427 (40.8%)	4424 (40.8%)	0.967	0.1
Metformin, *n* (%)	11,652 (28.3%)	3623 (30.4%)	< 0.001	4.5	3188 (29.4%)	3117 (28.7%)	0.288	1.4
Sulfonylureas, *n* (%)	6825 (16.6%)	1883 (15.8%)	0.037	2.2	1671 (15.4%)	1653 (15.2%)	0.734	0.5
GLP-1 receptor agonists, *n* (%)	1667 (4.1%)	490 (4.1%)	0.785	0.3	451 (4.2%)	456 (4.2%)	0.865	0.2
DPP4 inhibitors, *n* (%)	5081 (12.3%)	1733 (14.5%)	< 0.001	6.4	1473 (13.6%)	1460 (13.5%)	0.796	0.4
SGLT2 inhibitors, *n* (%)	2933 (7.1%)	949 (8%)	0.002	3.1	848 (7.8%)	838 (7.7%)	0.8	0.3
Thiazolidinediones, *n* (%)	1379 (3.4%)	585 (4.9%)	< 0.001	7.8	409 (3.8%)	492 (4.5%)	0.005	3.8
Antiplatelet therapy, *n* (%)	24,002 (58.3%)	5884 (49.3%)	< 0.001	18.1	5549 (51.1%)	5412 (49.9%)	0.063	2.5
Anticoagulant, *n* (%)	3706 (9%)	463 (3.9%)	< 0.001	21	524 (4.8%)	460 (4.2%)	0.037	2.8

ACE, angiotensin-converting enzyme; BP, blood pressure; COPD, chronic obstructive pulmonary disease; DPP4, dipeptidyl peptidase 4; GFR, glomerular filtration rate; GLP1, glucagon-like peptide-1; HDL, high-density lipoprotein; hsCRP, high sensitivity C-reactive protein; LDL, low-density lipoprotein; MDRD, Modification of Diet in Renal Disease formula; SGLT2, sodium glucose cotransporter-2; Std diff., standard difference.

### hsCRP Levels and Major Outcomes During Follow-Up in the Subgroup of Patients With Diabetes

**Figure 2:**
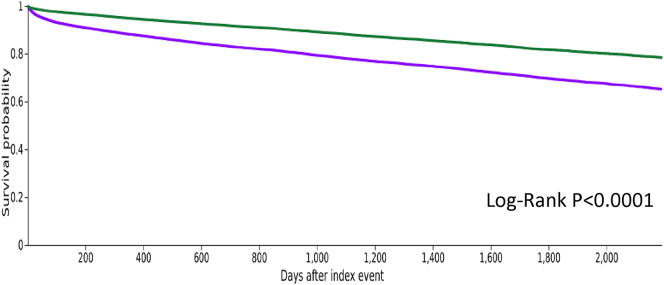
Event-free curve for all-cause death in the 2 groups. Event-free curve for all-cause death in matched patients with hsCRP < 2 mg/l (blue) versus patients with hsCRP ≥ 2 mg/l (red). hsCRP, high sensitivity C-reactive protein.

The median follow-up was 589 (interquartile range: 1366) days in patients with hsCRP ≥ 2 mg/l and 763 (1550) in patients with hsCRP < 2 mg/l. As shown in Table 4 and Figure 2, patients with hsCRP ≥ 2 mg/l had significant greater risks of all-cause death (yearly rate: 15.5% vs. 9.3%, HR: 1.899 [95% CI: 1.769–2.038]), ischemic strokes, thromboembolism, atrial fibrillation, cardiac arrest, myocardial infarction, and hospitalization for heart failure (Table 4). In addition, the rate of ESKD tended to be higher in patients with baseline hsCRP ≥ 2 mg/l versus ≤ 2 mg/l (HR: 1.508 [0.997–2.281], *P* = 0.05) (Table 4).

**Table 4:** Clinical outcomes during follow-up in the matched population of patients with diabetes

	hsCRP ≥ 2 mg/l		hsCRP < 2 mg/l		Hazard ratio (95% CI)	*P-*value
	(*n* = 10,850)		(*n* = 10,850)			
	Number of events	Yearly rate, %	Number of events	Yearly rate, %		
Death	2077	15.05	1225	9.30	1.899 (1.769–2.038)	< 0.0001
ESKD	53	0.47	39	0.34	1.508 (0.997–2.281)	0.05
MACE and other cardiovascular events						
Ischemic stroke or thromboembolism	74	0.65	38	0.30	2.186 (1.478–3.233)	< 0.0001
Acute MI	63	0.58	39	0.29	1.785 (1.197–2.663)	0.004
Atrial fibrillation	678	6.59	508	4.83	1.493 (1.331–1.675)	< 0.0001
VT/VF/cardiac arrest	416	3.16	338	2.24	1.331 (1.153–1.537)	< 0.0001
Cardiac arrest	170	1.37	94	0.66	1.979 (1.538–2.546)	< 0.0001
VT or VF	277	2.06	275	1.81	1.084 (0.917–1.281)	0.34
MI/stroke/heart failure/death	2659	17.53	1562	10.91	1.916 (1.800–2.040)	< 0.0001
MI, ischemic stroke, or heart failure	753	4.47	400	2.31	2.013 (1.783–2.273)	< 0.0001
Incident heart failure	934	10.64	861	9.40	1.212 (1.105–1.330)	< 0.0001
Hospitalization for heart failure	415	2.50	235	1.38	1.875 (1.597–2.200)	< 0.0001

CI, confidence interval; ESKD, end-stage kidney disease; hsCRP, high sensitivity C-reactive protein; MACE, major cardiovascular event; MI, myocardial infarction; VF, ventricular fibrillation; VT, ventricular tachycardia.

### Baseline Characteristics Before and After Propensity Score Matching Among Patients With No Diabetes

Overall, 80,266 patients without diabetes had CKD and hsCRP results (62,763 patients with hsCRP ≥ 2 mg/l and 17,503 patients with hsCRP < 2 mg/l) (Supplementary Table S1**)**. Before propensity score matching, most parameters were different between the 2 groups (Supplementary Table S1). After propensity score matching, 16,530 patients with hsCRP ≥ 2 mg/l were compared with 16,530 patients with hsCRP < 2 mg/l (Supplementary Table S1). These 2 groups were well-matched (standard difference of all parameters between the 2 groups < 10% for almost all parameters) on baseline characteristics (Supplementary Table S1).

### hsCRP Levels and Major Outcomes During Follow-Up in the Subgroup of Patients With No Diabetes

The median follow-up was 452 (interquartile range: 1220) days in patients with hsCRP ≥ 2 mg/l and 553 (1332) in patients with hsCRP < 2 mg/l. As shown in Supplementary Table S2, patients with hsCRP ≥ 2 mg/l had significant greater risks of all-cause death (yearly rate: 17% vs. 11%, HR: 1.965 [95% CI: 1.849–2.090]), ischemic strokes, thromboembolism, atrial fibrillation, cardiac arrest, myocardial infarction, and hospitalization for heart failure (Supplementary Table S2). The risk of ESKD did not reach the statistical threshold (HR: 1.247 [0.653–2.382], *P* = 0.5) (Supplementary Table 2).

## Discussion

In the present study conducted in a large population of well-matched patients with CKD stage 3 and 4 or albuminuria (A3) (median eGFR: 52 ml/min per 1.73 m^2^, albumin-to-creatinine ratio: 210 mg/g), we found that hsCRP ≥ 2 mg/l was associated with significantly higher risks of all-cause death and MACE compared with patients with hsCRP < 2 mg/l, and among them, in those with no diabetes mellitus. Similar results were found for MACE and death risks among patients with diabetes and hsCRP ≥ 2 mg/l, but in addition, the risk of ESKD was higher than in patients with hsCRP < 2 mg/l.

The association between a baseline hsCRP level ≥ 2 mg/l with increased risks of a wide range of major cardiovascular and cerebrovascular events, ventricular and atrial arrythmias, and all-cause death among patients with CKD was observed, though these patients were well-matched for traditional cardiovascular risk factors, including comorbid conditions, a wide range of biological and clinical parameters and baseline treatments. Whether this association between CRP levels and cardiovascular events is causal has been extensively discussed and is still debated. Mendelian randomization studies in 2 cohorts and a meta-analysis of 47 studies (194,418 subjects) did not find an independent association between CRP levels and coronary heart disease.^9^ In marked contrast, in a recent genome-wide association in half a million subjects, a genetic risk score of CRP was associated with coronary heart disease.^10^ A recent study from the UK biobank indicated that CRP levels > 10 mg/l (vs. CRP < 0.5 mg/l) were significantly and positively associated with the risk of incident atrial fibrillation.^11^ Furthermore, they found a continuous positive relationship between baseline CRP levels and incident ventricular arrythmia.^11^ CRP may promote inflammation and subsequent myocardial fibrosis through the TLR4/NF-κB/TGF-β pathway.^12^ Another study showed that CRP played a proarrhythmic role by directly affecting calcium homeostasis in cardiomyocytes.^13^ These studies were mainly conducted in the general population. Our study included patients with CKD and albuminuria. These associations were confirmed in the subgroup of patients with diabetes mellitus. Our results are in accordance with smaller observational studies. In 543 patients with CKD stage 5, markers of inflammation, including elevated CRP were associated with baseline cardiovascular disease and mortality during follow-up.^14^ In 2399 patients with mainly CKD stage 3a or 3b, markers of inflammation, including elevated CRP were associated with baseline cardiovascular disease and mortality during follow-up.^5^ An independent association was found in CKD populations and the risk of death,^15^ including in those with coronary artery disease.^16^^,^^17^

We observed that hsCRP ≥ 2 mg/l (vs. hsCRP < 2 mg/l) was associated with an increased risk of ESKD in subjects with diabetes mellitus, but not in the whole population. The absence of a significant association between elevated hsCRP levels and the risk of ESKD in the whole population was probably because of a lack of power, because very few patients reached ESKD. It was found that hsCRP predicted the risk of developing CKD in a population-based cohort.^18^ A faster progression to kidney failure associated with elevated hsCRP was noted in another study.^5^ It was therefore proposed by the authors that “traditional cardiovascular risk estimates could be improved by adding markers of inflammation and measures of kidney function.”^5^

Whether targeting inflammation is beneficial or detrimental in patients with risk of ESKD and MACE is unclear. Statins have demonstrated antiinflammatory effects.^19^ However, a recent meta-analysis found no association with CKD progression toward ESKD.^20^ Targeting interleukin-1β using canakinumab decreased hsCRP and provided cardioprotection, even in a subgroup of patients with mild CKD; however, no nephroprotection was found in a recent clinical trial.^21^ In a phase 2 trial, ziltivekimab, a monoclonal antibody directed against the interleukin-6 ligand, markedly reduced hsCRP in patients with stages 3 and 4 CKD.^8^ The randomized clinical trial titled “ZEUS-Effects of Ziltivekimab Versus Placebo on Cardiovascular Outcomes in Participants With Established Atherosclerotic Cardiovascular Disease, Chronic Kidney Disease and Systemic Inflammation” is ongoing.^22^ This study in 6000 patients with CKD will analyze whether ziltivekimab results in cardioprotection and effective nephroprotection.^22^ Interestingly, the inclusion criteria for this study are similar to those used in the present cohort. Although it is tempting to speculate that similar results would be observed in the ZEUS clinical trial, they cannot be extrapolated to ZEUS future findings.

The study has limitations. A selection bias is possible, although a propensity matching score was used. It is a real-life study, and therefore, even after careful propensity score matching, differences in the management of these subjects could still be present. We focused on analysis on subjects with mild to moderate KDIGO CKD 3 and 4 or albuminuria > 200 mg/g (KDIGO A3), that is, patients who have the highest risk of progression of renal diseases. It is possible that different results could be found in subjects with more advanced renal dysfunction. We believe that it is unlikely because smaller observational studies confirmed that elevated CRP is present in patients with CKD, regardless of the CKD stage, and that cardiovascular events were associated with elevated hsCRP in mild CKD stages as well as in patients under chronic dialysis.

The strength of this study derives from its size and design. Overall, after propensity score matching, 55,160 subjects for the whole population and 21,700 subjects with diabetes mellitus were evaluated (from an initial population of 163,854 subjects). The 2 subgroups of subjects were well-matched on all traditional cardiovascular risk factors, including blood pressure; comorbid conditions; LDL cholesterol and high-density lipoprotein cholesterol levels; hemoglobin A1c; eGFR and albuminuria; and baseline treatments, including sodium-glucose cotransporter-2 inhibitors and glucagon-like peptide-1 receptor agonists for subjects with diabetes mellitus. Careful analyses were performed in the whole population and in subjects with diabetes mellitus. Cardiovascular outcomes, including atrial and ventricular arrythmias, were thoroughly evaluated and hard renal outcomes were assessed.

In conclusion, the presence of systemic inflammation (defined as hsCRP ≥ 2 mg/l) is associated with elevated risk of ischemic stroke, MACE, atrial and ventricular arrythmias among subjects with mild CKD 3 and 4 or elevated albuminuria (A3), independently of traditional cardiovascular risk factors and treatments, even in the absence of diabetes mellitus. In addition, hsCRP ≥ 2 mg/l tended to be associated with a greater risk of ESKD, independently of baseline blood pressure, renal function, and treatments in the subgroup of patients with diabetes mellitus.

## Disclosure

All the authors declared no competing interests.

## Author Contributions

JMH and LF contributed to study conception, statistical analysis, writing of the first draft, and access to the data. VM, JBdF, SR, AB, and SC contributed to corrections of the drafts and interpretation of data. All the authors contributed to the drafting and correction of the manuscript.

## Supplementary Material

Supplementary File (PDF) Propensity score matching. **Table S1.** Baseline characteristics of patients with no diabetes before and after propensity score matching. **Table S2.** Clinical outcomes during follow-up in the matched population of patients with no diabetes. Strobe checklist.

## Footnotes

Supplementary File (PDF)
Propensity score matching. **Table S1.** Baseline characteristics of patients with no diabetes before and after propensity score matching. **Table S2.** Clinical outcomes during follow-up in the matched population of patients with no diabetes. Strobe checklist.
