# Corrigendum to “ESKD, Major Cardiovascular Events, and Death Associated With Systemic Inflammation” [*Kidney International Reports* Volume 11, Issue 4, April 2026, 106364]

**DOI:** 10.1016/j.ekir.2026.106660

**Published:** 2026-06-04

**Authors:** Jean-Michel Halimi, Valentin Maisons, Jean-Baptiste de Fréminville, Sébastien Roger, Arnaud Bisson, Stéphanie Chadet, Laurent Fauchier

**Affiliations:** 1 Service de Néphrologie-Hypertension, Dialyse, Transplantation rénale, CHU de Tours, Tours, France; 2 INSERM UMR1327, ISCHEMIA, Université de Tours, Tours, France; 3 INI-CRCT, Tours, France; 4 INSERM UMR1246, SPHERE, Université de Tours, Université de Nantes, Tours, Nantes, France; 5 Service de Cardiologie, CHU de Tours, Tours, France

The authors have corrected the Supplementary Materials file in the above article, which can be accessed here.

The reported standard deviation for hsCRP in the hsCRP<2 mg/L group appeared unexpectedly large. This was related to an issue in the initial TriNetX query configuration and has now been corrected in the tables in the Supplementary Materials.

DOI of original article: https://doi.org/10.1016/j.ekir.2026.106364

