# Supplementary material for: ESKD, Major Cardiovascular Events, and Death Associated With Systemic Inflammation
Source: Kidney Int Rep. 2026 Feb 17;11(4):106364. doi: 10.1016/j.ekir.2026.106364 (PMC12995490; doi:10.1016/j.ekir.2026.106364)
Supplement: Supplementary File (PDF) — Propensity score matching. Table S1. Baseline characteristics of patients with no diabetes before and after propensity score matching. Table S2. Clinical outcomes during follow-up in the matched population of patients with no diabetes. Strobe checklist. [file mmc1.pdf]

Supplementary material

Propensity score matching in the whole population, in patients with diabetes and in those with no diabetes.

Propensity score matching was performed on 57 characteristic(s). In the Demographics category patients were matched on Age at Index, Male, Hispanic or Latino, White, Black or African American, Asian, Unknown Race characteristic(s). In the Diagnosis category patients were matched on Essential (primary) hypertension, Acute myocardial infarction, Cerebral infarction, Diabetes mellitus, Overweight, obesity and other hyperalimentation, Disorders of thyroid gland, Malnutrition, Disorders of lipoprotein metabolism and other lipidemias, Heart failure, Nonrheumatic mitral valve disorders, Acute kidney failure and chronic kidney disease, Chronic obstructive pulmonary disease, unspecified, Diseases of the respiratory system, Sleep apnea, Peripheral vascular disease, unspecified, Personal history of nicotine dependence, Dilated cardiomyopathy, Atrial fibrillation and flutter, Presence of cardiac pacemaker, Alcohol abuse, Neoplasms, Alzheimer's disease, Anxiety, dissociative, stress-related, somatoform and other nonpsychotic mental disorders, Mood [affective] disorders, Other anemias, Long term (current) use of anticoagulants, Ischemic heart diseases characteristic(s). In the Medication category patients were matched on beta blockers/related, antilipemic agents, diuretics, antiarrythmics, calcium-channel blockers, angiotensin converting-enzyme inhibitors, angiotensin II blockers, drugs used in diabetes, insulins and analogues, biguanides, sulfonylureas, glucagon-like peptide-1 analogues, dipeptidyl peptidase 4 inhibitors, Sodium-glucose co-transporter 2 inhibitors, platelet aggregation inhibitors. In the Laboratory category patients were matched on total cholesterol, LDL-cholesterol, HDL-cholesterol, hemoglobin A1c (%), BMI, blood pressure, estimated glomerular filtration rate (Creatinine-based MDRD formula), Hemoglobin levels.

Supplemental Table 1. Baseline characteristics of patients with no diabetes before and after propensity score matching.

	Before propensity-score matching				After propensity-score matching			
	ZEUS no DM, CRP sup 2 (n = 62,763)	ZEUS no DM, CRP inf 2 (n = 17,503)	P-Value	Std diff. (%)	ZEUS no DM, CRP sup 2 (n = 16,530)	ZEUS no DM, CRP inf 2 (n = 16,530)	P-Value	Std diff. (%)
Age, n (%)	73.8 +/- 12.1	71.9 +/- 11.8	<0.001	15.6	73.1 +/- 12.1	72.3 +/- 11.8	<0.001	6.4
Men, n (%)	32699 (52.1%)	9290 (53.1%)	0.022	2	8636 (52.2%)	8769 (53%)	0.143	1.6
Systolic BP (mm Hg), mean±SD	125.5 +/- 24.1	129.4 +/- 23.4	<0.001	16.6	127.3 +/- 23.7	129.4 +/- 23.4	<0.001	8.9
Diastolic BP (mm Hg), mean±SD	69.1 +/- 14.6	71.6 +/- 13.3	<0.001	17.9	70.4 +/- 14.4	71.6 +/- 13.3	<0.001	8.7
Body mass index (kg/m2), mean±SD	27.9 +/- 6.5	26.3 +/- 5.1	<0.001	27.3	26.9 +/- 5.6	26.5 +/- 5.2	<0.001	7.2
White, n (%)	26418 (42.1%)	7370 (42.1%)	0.971	0	7166 (43.4%)	7320 (44.3%)	0.088	1.9
Black or African American, n (%)	3187 (5.1%)	614 (3.5%)	<0.001	7.8	582 (3.5%)	614 (3.7%)	0.346	1
Asian, n (%)	2527 (4%)	2618 (15%)	<0.001	38	1894 (11.5%)	1702 (10.3%)	0.001	3.7
Hispanic or Latino, n (%)	1348 (2.1%)	221 (1.3%)	<0.001	6.8	209 (1.3%)	220 (1.3%)	0.593	0.6
Unknown Race, n (%)	29351 (46.8%)	6513 (37.2%)	<0.001	19.4	6533 (39.5%)	6509 (39.4%)	0.787	0.3
**Comorbid conditions**								
Hypertension, n (%)	31272 (49.8%)	8879 (50.7%)	0.035	1.8	8350 (50.5%)	8350 (50.5%)	1	0
Smoker, n (%)	5559 (8.9%)	1052 (6%)	<0.001	10.9	1076 (6.5%)	1042 (6.3%)	0.445	0.8
Overweight or obesity, n (%)	5613 (8.9%)	1105 (6.3%)	<0.001	9.9	1077 (6.5%)	1094 (6.6%)	0.706	0.4
Dyslipidaemia, n (%)	21299 (33.9%)	7209 (41.2%)	<0.001	15	6692 (40.5%)	6659 (40.3%)	0.711	0.4
Alcohol related diagnoses, n (%)	1145 (1.8%)	209 (1.2%)	<0.001	5.2	215 (1.3%)	208 (1.3%)	0.732	0.4
Heart failure, n (%)	13847 (22.1%)	2639 (15.1%)	<0.001	18	2646 (16%)	2568 (15.5%)	0.239	1.3
Coronary artery disease, n (%)	36730 (58.5%)	10820 (61.8%)	<0.001	6.7	10230 (61.9%)	10223 (61.8%)	0.937	0.1
Myocardial infarction, n (%)	4843 (7.7%)	1555 (8.9%)	<0.001	4.2	1471 (8.9%)	1491 (9%)	0.7	0.4
Dilated cardiomyopathy, n (%)	898 (1.4%)	184 (1.1%)	<0.001	3.4	185 (1.1%)	171 (1%)	0.456	0.8
Ischemic stroke, n (%)	16037 (25.6%)	3953 (22.6%)	<0.001	6.9	3784 (22.9%)	3771 (22.8%)	0.865	0.2
Intracranial hemorrhage, n (%)	1221 (1.9%)	302 (1.7%)	0.059	1.6	337 (2%)	283 (1.7%)	0.029	2.4
Atrial fibrillation or flutter, n (%)	12818 (20.4%)	2940 (16.8%)	<0.001	9.3	2873 (17.4%)	2818 (17%)	0.423	0.9
Kidney disease, n (%)	13565 (21.6%)	2860 (16.3%)	<0.001	13.5	2846 (17.2%)	2703 (16.4%)	0.035	2.3
Lung disease, n (%)	23266 (37.1%)	5403 (30.9%)	<0.001	13.1	5214 (31.5%)	5148 (31.1%)	0.434	0.9
COPD, n (%)	5475 (8.7%)	1054 (6%)	<0.001	10.4	1072 (6.5%)	1023 (6.2%)	0.269	1.2

Sleep apnoea syndrome, n (%)	3925 (6.3%)	1078 (6.2%)	0.647	0.4	1039 (6.3%)	1036 (6.3%)	0.946	0.1
Peripheral vascular disease, n (%)	5013 (8%)	1003 (5.7%)	<0.001	8.9	1019 (6.2%)	983 (5.9%)	0.406	0.9
Previous cancer, n (%)	13902 (22.1%)	3990 (22.8%)	0.069	1.5	3795 (23%)	3703 (22.4%)	0.227	1.3
Anemia, n (%)	6717 (10.7%)	1529 (8.7%)	<0.001	6.6	1542 (9.3%)	1479 (8.9%)	0.229	1.3
**Biology measurements**								
Total cholesterol (mg/dL), mean±SD	162.7 +/- 49.4	164.7 +/- 47.3	<0.001	4.1	165.6 +/- 47.8	163.8 +/- 47.6	0.005	3.8
LDL cholesterol (mg/dL), mean±SD	91.7 +/- 37.8	90.1 +/- 37.6	<0.001	4.3	91.9 +/- 37.0	89.4 +/- 37.8	<0.001	6.8
HDL cholesterol (mg/dL), mean±SD	49.0 +/- 17.5	52.5 +/- 18.0	<0.001	19.5	51.2 +/- 17.1	52.3 +/- 18.2	<0.001	5.9
Triglyceride (mg/dL), mean±SD	125.4 +/- 139.7	122.8 +/- 226.3	0.155	1.4	124.6 +/- 146.5	122.9 +/- 227.9	0.53	0.9
Hemoglobin A1c (%), mean±SD	5.8 +/- 1.0	5.7 +/- 0.9	<0.001	14.4	5.7 +/- 0.8	5.6 +/- 0.9	<0.001	7.8
Estimated GFR (MDRD, ml/min), mean±SD	51.1 +/- 18.4	54.2 +/- 15.1	<0.001	18.2	53.0 +/- 17.0	53.9 +/- 15.2	<0.001	6
Albuminuria (mg/g), mean±SD	152.8 +/- 501.8	237.9 +/- 1085.3	0.042	10.1	161.4 +/- 532.4	240.7 +/- 1092.6	0.236	9.2
Albuminuria >200 mg/g, n (%)	201 (0.3%)	40 (0.2%)	0.05	1.8	49 (0.3%)	40 (0.2%)	0.339	1.1
CRP (mg/L), mean±SD	15.1 +/- 25.5	1.0 +/- 7.4	<0.001	75.4	12.9 +/- 22.2	1.0 +/- 7.6	<0.001	72.1
Hemoglobin (g/dl), mean±SD	12.3 +/- 2.4	13.2 +/- 2.1	<0.001	38.2	12.7 +/- 2.3	13.1 +/- 2.1	<0.001	19.3
**Baseline treatments**								
Beta Blockers, n (%)	30961 (49.3%)	8001 (45.7%)	<0.001	7.2	7675 (46.4%)	7723 (46.7%)	0.597	0.6
Calcium Channel Blockers, n (%)	19965 (31.8%)	5607 (32%)	0.573	0.5	5243 (31.7%)	5177 (31.3%)	0.435	0.9
ACE Inhibitors, n (%)	17813 (28.4%)	4587 (26.2%)	<0.001	4.9	4416 (26.7%)	4497 (27.2%)	0.315	1.1
Angiotensin II Inhibitors, n (%)	13863 (22.1%)	4424 (25.3%)	<0.001	7.5	4054 (24.5%)	4004 (24.2%)	0.522	0.7
Digitalis glycosides, n (%)	2756 (4.4%)	491 (2.8%)	<0.001	8.5	571 (3.5%)	468 (2.8%)	0.001	3.6
Diuretics, n (%)	28002 (44.6%)	6305 (36%)	<0.001	17.6	6127 (37.1%)	6091 (36.8%)	0.682	0.5
Lipid lowering drugs, n (%)	32533 (51.8%)	9668 (55.2%)	<0.001	6.8	9017 (54.5%)	9088 (55%)	0.433	0.9
Antiplatelet therapy, n (%)	32996 (52.6%)	9034 (51.6%)	0.025	1.9	8635 (52.2%)	8672 (52.5%)	0.684	0.4
Anticoagulant, n (%)	4437 (7.1%)	723 (4.1%)	<0.001	12.8	757 (4.6%)	717 (4.3%)	0.286	1.2

Supplemental Table 2. Clinical outcomes during follow-up in the matched population of patients with no diabetes.

	ZEUS no DM, CRP sup 2 (n = 16,530)		ZEUS no DM, CRP inf 2 (n = 16,530)			
	**Number of events**	**Yearly rate, %**	**Number of events**	**Yearly rate, %**	**Hazard ratio (95% CI)**	**p value**
**Death**	2884	16.85	1586	10.57	1.965 (1.849-2.090)	<0.0001
**ESKD**	20	0.12	17	0.12	1.247 (0.653-2.382)	0.5
**MACE or other cardiovascular events**						
Ischemic stroke or thromboembolism	109	0.75	79	0.55	1.500 (1.123-2.004)	0.01
Acute MI	88	0.58	62	0.45	1.502 (1.085-2.079)	0.01
AF	940	7.58	713	5.78	1.430 (1.298-1.577)	<0.0001
Incident hypertension	1011	15.96	840	13.92	1.343 (1.226-1.472)	<0.0001
VT/VF/Cardiac arrest	660	3.74	508	2.79	1.363 (1.214-1.530)	<0.0001
Cardiac arrest	309	1.70	132	0.78	2.450 (1.998-3.004)	<0.0001
VT/VF	378	2.27	397	2.14	1.001 (0.869-1.152)	0.99
MI/stroke/HF/death	3858	20.14	2461	13.89	1.688 (1.605-1.775)	<0.0001
MI, ischemic stroke or HF	1229	5.76	982	4.53	1.300 (1.195-1.413)	<0.0001
Incident HF	1202	9.41	806	6.02	1.620 (1.482-1.771)	<0.0001
Hosp. for HF	539	2.95	409	2.04	1.379 (1.212-1.568)	<0.0001

STROBE Statement—checklist of items that should be included in reports of observational studies

	Item No	Recommendation
**Title and abstract**	1	(*a*) Indicate the study's design with a commonly used term in the title or the abstract
		(*b*) Provide in the abstract an informative and balanced summary of what was done and what was found
**Introduction**		
Background/rationale	2	Explain the scientific background and rationale for the investigation being reported
Objectives	3	State specific objectives, including any prespecified hypotheses
**Methods**		
Study design	4	Present key elements of study design early in the paper
Setting	5	Describe the setting, locations, and relevant dates, including periods of recruitment, exposure, follow-up, and data collection
Participants	6	(*a*) *Cohort study*—Give the eligibility criteria, and the sources and methods of selection of participants. Describe methods of follow-up<br>*Case-control study*—Give the eligibility criteria, and the sources and methods of case ascertainment and control selection. Give the rationale for the choice of cases and controls<br>*Cross-sectional study*—Give the eligibility criteria, and the sources and methods of selection of participants
		(*b*) *Cohort study*—For matched studies, give matching criteria and number of exposed and unexposed<br>*Case-control study*—For matched studies, give matching criteria and the number of controls per case
Variables	7	Clearly define all outcomes, exposures, predictors, potential confounders, and effect modifiers. Give diagnostic criteria, if applicable
Data sources/ measurement	8*	For each variable of interest, give sources of data and details of methods of assessment (measurement). Describe comparability of assessment methods if there is more than one group
Bias	9	Describe any efforts to address potential sources of bias
Study size	10	Explain how the study size was arrived at
Quantitative variables	11	Explain how quantitative variables were handled in the analyses. If applicable, describe which groupings were chosen and why
Statistical methods	12	(*a*) Describe all statistical methods, including those used to control for confounding
		(*b*) Describe any methods used to examine subgroups and interactions
		(*c*) Explain how missing data were addressed
		(*d*) *Cohort study*—If applicable, explain how loss to follow-up was addressed<br>*Case-control study*—If applicable, explain how matching of cases and controls was addressed<br>*Cross-sectional study*—If applicable, describe analytical methods taking account of sampling strategy
		(*e*) Describe any sensitivity analyses

Continued on next page

Results		
Participants	13*	(a) Report numbers of individuals at each stage of study—eg numbers potentially eligible, examined for eligibility, confirmed eligible, included in the study, completing follow-up, and analysed
		(b) Give reasons for non-participation at each stage
		(c) Consider use of a flow diagram
Descriptive data	14*	(a) Give characteristics of study participants (eg demographic, clinical, social) and information on exposures and potential confounders
		(b) Indicate number of participants with missing data for each variable of interest
		(c) *Cohort study*—Summarise follow-up time (eg, average and total amount)
Outcome data	15*	*Cohort study*—Report numbers of outcome events or summary measures over time
		Case-control study—Report numbers in each exposure category, or summary measures of exposure
		Cross-sectional study—Report numbers of outcome events or summary measures
Main results	16	(*a*) Give unadjusted estimates and, if applicable, confounder-adjusted estimates and their precision (eg, 95% confidence interval). Make clear which confounders were adjusted for and why they were included
		(*b*) Report category boundaries when continuous variables were categorized
		(*c*) If relevant, consider translating estimates of relative risk into absolute risk for a meaningful time period
Other analyses	17	Report other analyses done—eg analyses of subgroups and interactions, and sensitivity analyses
**Discussion**		
Key results	18	Summarise key results with reference to study objectives
Limitations	19	Discuss limitations of the study, taking into account sources of potential bias or imprecision. Discuss both direction and magnitude of any potential bias
Interpretation	20	Give a cautious overall interpretation of results considering objectives, limitations, multiplicity of analyses, results from similar studies, and other relevant evidence
Generalisability	21	Discuss the generalisability (external validity) of the study results
**Other information**		
Funding	22	Give the source of funding and the role of the funders for the present study and, if applicable, for the original study on which the present article is based

*Give information separately for cases and controls in case-control studies and, if applicable, for exposed and unexposed groups in cohort and cross-sectional studies.

Note: An Explanation and Elaboration article discusses each checklist item and gives methodological background and published examples of transparent reporting. The STROBE checklist is best used in conjunction with this article (freely available on the Web sites of PLoS Medicine at http://www.plosmedicine.org/, Annals of Internal Medicine at http://www.annals.org/, and Epidemiology at http://www.epidem.com/). Information on the STROBE Initiative is available at www.strobe-statement.org.
