# Supplementary material for: Corrigendum to “ESKD, Major Cardiovascular Events, and Death Associated With Systemic Inflammation” [Kidney International Reports Volume 11, Issue 4, April 2026, 106364]
Source: Kidney Int Rep. 2026 Jun 4;11(8):106660. doi: 10.1016/j.ekir.2026.106660 (PMC13315682; doi:10.1016/j.ekir.2026.106660)
Supplement: Supplementary Material [file mmc1.pdf]

Table 1. Baseline characteristics of patients before and after propensity score matching.

	Before propensity-score matching			After propensity-score matching		
	hsCRP ≥ 2 mg/L (n = 107717)	hsCRP < 2 mg/L (n = 16357)	Std diff. (%)	hsCRP ≥ 2 mg/L (n = 15880)	hsCRP < 2 mg/L (n = 15880)	Std diff. (%)
**Age at Index, n (%)**	74.4 +/- 11.4	73.4 +/- 11.2	8.4	74.4 +/- 11.6	73.5 +/- 11.2	8.5
**Men, n (%)**	59064 (54.8%)	8374 (51.2%)	7.3	8034 (50.6%)	8141 (51.3%)	1.3
**Systolic BP (mm Hg), mean±SD**	130.1 +/- 23.0	134.0 +/- 22.4	16.9	132.5 +/- 22.6	133.9 +/- 22.4	6.2
**Diastolic BP (mm Hg), mean±SD**	69.4 +/- 13.6	73.0 +/- 13.2	26.6	71.5 +/- 13.4	72.9 +/- 13.2	10.8
**Body mass index (kg/m2), mean±SD**	29.5 +/- 7.2	27.3 +/- 5.5	33.6	27.7 +/- 6.2	27.4 +/- 5.5	4.8
**White, n (%)**	55784 (51.8%)	6284 (38.4%)	27.1	6093 (38.4%)	6250 (39.4%)	2
**Black or African American, n (%)**	12717 (11.8%)	1266 (7.7%)	13.7	1333 (8.4%)	1264 (8%)	1.6
**Asian, n (%)**	4808 (4.5%)	2647 (16.2%)	39.3	2355 (14.8%)	2243 (14.1%)	2
**Hispanic or Latino, n (%)**	3747 (3.5%)	294 (1.8%)	10.5	290 (1.8%)	294 (1.9%)	0.2
**Unknown race, n (%)**	31092 (28.9%)	5777 (35.3%)	13.9	5707 (35.9%)	5744 (36.2%)	0.5
Comorbid conditions						
**Hypertension, n (%)**	75984 (70.5%)	9850 (60.2%)	21.8	9652 (60.8%)	9596 (60.4%)	0.7
**Diabetes mellitus, n (%)**	48953 (45.4%)	5820 (35.6%)	20.2	5565 (35%)	5637 (35.5%)	0.9
**Smoker, n (%)**	23923 (22.2%)	1815 (11.1%)	30.2	1833 (11.5%)	1811 (11.4%)	0.4
**Overweight or obesity, n (%)**	22405 (20.8%)	1594 (9.7%)	31.1	1642 (10.3%)	1589 (10%)	1.1
**Dyslipidaemia, n (%)**	63345 (58.8%)	8456 (51.7%)	14.3	8300 (52.3%)	8178 (51.5%)	1.5
**Alcohol related diagnoses, n (%)**	2177 (2%)	141 (0.9%)	9.7	144 (0.9%)	141 (0.9%)	0.2
**Heart failure, n (%)**	38198 (35.5%)	3089 (18.9%)	37.9	3107 (19.6%)	3057 (19.3%)	0.8
**Coronary artery disease, n (%)**	72634 (67.4%)	9721 (59.4%)	16.7	9549 (60.1%)	9450 (59.5%)	1.3
**Myocardial infarction, n (%)**	12952 (12%)	1224 (7.5%)	15.4	1245 (7.8%)	1204 (7.6%)	1
**Dilated cardiomyopathy, n (%)**	2721 (2.5%)	182 (1.1%)	10.6	195 (1.2%)	177 (1.1%)	1.1
**Ischemic stroke, n (%)**	24664 (22.9%)	4126 (25.2%)	5.4	3952 (24.9%)	4006 (25.2%)	0.8
**Intracranial hemorrhage, n (%)**	1924 (1.8%)	275 (1.7%)	0.8	295 (1.9%)	269 (1.7%)	1.2
**Atrial fibrillation or flutter, n (%)**	29901 (27.8%)	2635 (16.1%)	28.4	2629 (16.6%)	2601 (16.4%)	0.5
**Kidney disease, n (%)**	57410 (53.3%)	5353 (32.7%)	42.5	5264 (33.1%)	5218 (32.9%)	0.6
**Lung disease, n (%)**	57899 (53.8%)	5476 (33.5%)	41.8	5386 (33.9%)	5415 (34.1%)	0.4
**COPD, n (%)**	17984 (16.7%)	1278 (7.8%)	27.3	1259 (7.9%)	1269 (8%)	0.2
**Sleep apnoea syndrome, n (%)**	15459 (14.4%)	1290 (7.9%)	20.7	1289 (8.1%)	1276 (8%)	0.3
**Peripheral vascular disease, n (%)**	19211 (17.8%)	1464 (9%)	26.3	1486 (9.4%)	1455 (9.2%)	0.7
**Previous cancer, n (%)**	27797 (25.8%)	3688 (22.5%)	7.6	3567 (22.5%)	3585 (22.6%)	0.3
**Anemia, n (%)**	26495 (24.6%)	2364 (14.5%)	25.8	2445 (15.4%)	2320 (14.6%)	2.2

Biology measurements						
**Total cholesterol (mg/dL), mean±SD**	155.7 +/- 48.0	162.0 +/- 47.4	13.3	164.4 +/- 46.1	161.7 +/- 47.8	5.8
**LDL cholesterol (mg/dL), mean±SD**	84.0 +/- 38.2	86.9 +/- 37.1	7.8	89.5 +/- 36.7	86.7 +/- 37.3	7.5
**HDL cholesterol (mg/dL), mean±SD**	43.0 +/- 17.0	49.5 +/- 18.1	37.1	48.2 +/- 17.2	49.3 +/- 18.2	6.2
**Triglyceride (mg/dL), mean±SD**	140.4 +/- 104.5	130.8 +/- 91.0	9.8	133.4 +/- 92.6	131.1 +/- 91.5	2.5
**Hemoglobin A1c (%), mean±SD**	6.8 +/- 1.8	6.3 +/- 1.6	28.9	6.5 +/- 1.6	6.3 +/- 1.6	13.2
**Estimated GFR (MDRD, ml/min), mean±SD**	45.7 +/- 20.4	49.9 +/- 15.9	23	49.3 +/- 18.0	49.7 +/- 15.9	2.8
**Albuminuria (mg/g), mean±SD**	1593.0 +/- 12831.3	1594.5 +/- 12586.0	0	1096.4 +/- 8811.4	1625.0 +/- 12724.7	4.8
**Albuminuria >=200 mg/g, n (%)**	2070 (1.9%)	244 (1.5%)	3.3	267 (1.7%)	239 (1.5%)	1.4
**hsCRP (mg/L), mean±SD**	53.4 +/- 70.8	0.8 +/- 0.5	105	40.1 +/- 61.0	0.8 +/- 0.5	90.9
**Hemoglobin (g/dl), mean±SD**	11.7 +/- 2.3	12.9 +/- 2.0	54.4	12.5 +/- 2.1	12.9 +/- 2.0	19.8
Baseline treatments						
**Beta Blockers, n (%)**	63579 (59%)	7369 (45.1%)	28.2	7197 (45.3%)	7189 (45.3%)	0.1
**Calcium Channel Blockers, n (%)**	45388 (42.1%)	5773 (35.3%)	14.1	5656 (35.6%)	5570 (35.1%)	1.1
**ACE Inhibitors, n (%)**	29858 (27.7%)	3422 (20.9%)	15.9	3409 (21.5%)	3375 (21.3%)	0.5
**Angiotensin II Inhibitors, n (%)**	28681 (26.6%)	4231 (25.9%)	1.7	4088 (25.7%)	4059 (25.6%)	0.4
**Digitalis glycosides, n (%)**	4674 (4.3%)	345 (2.1%)	12.6	417 (2.6%)	338 (2.1%)	3.3
**Diuretics, n (%)**	56020 (52%)	5465 (33.4%)	38.3	5373 (33.8%)	5381 (33.9%)	0.1
**Lipid lowering drugs, n (%)**	66267 (61.5%)	8718 (53.3%)	16.7	8504 (53.6%)	8454 (53.2%)	0.6
**Glucose-lowering therapy, n (%)**	46728 (43.4%)	4713 (28.8%)	30.7	4657 (29.3%)	4596 (28.9%)	0.8
**Insulin, n (%)**	39527 (36.7%)	3164 (19.3%)	39.4	3250 (20.5%)	3134 (19.7%)	1.8
**Non-insulin glucose-lowering therapy, n (%)**	25419 (23.6%)	3169 (19.4%)	10.3	3036 (19.1%)	3058 (19.3%)	0.4
**Metformin, n (%)**	14510 (13.5%)	2092 (12.8%)	2	1979 (12.5%)	2006 (12.6%)	0.5
**Sulfonylureas, n (%)**	10888 (10.1%)	1290 (7.9%)	7.8	1247 (7.9%)	1242 (7.8%)	0.1
**GLP-1 receptor agonists, n (%)**	2755 (2.6%)	321 (2%)	4	313 (2%)	316 (2%)	0.1
**DPP4 inhibitors, n (%)**	8258 (7.7%)	1204 (7.4%)	1.2	1131 (7.1%)	1145 (7.2%)	0.3
**SGLT2 inhibitors, n (%)**	3676 (3.4%)	495 (3%)	2.2	458 (2.9%)	474 (3%)	0.6
**Thiazolidinediones, n (%)**	1632 (1.5%)	316 (1.9%)	3.2	224 (1.4%)	292 (1.8%)	3.4
**Antiplatelet therapy, n (%)**	64539 (59.9%)	8272 (50.6%)	18.9	8090 (50.9%)	8037 (50.6%)	0.7
**Anticoagulant, n (%)**	16654 (15.5%)	1113 (6.8%)	27.8	1145 (7.2%)	1108 (7%)	0.9

Table 2. Clinical outcomes during follow-up in the matched population (follow-up 3.1 ± 2.2 years, median 3, IQR 4.5)

	hsCRP ≥ 2 mg/L (n = 15880)		hsCRP < 2 mg/L (n = 15880)				
	**Number of events**	**Yearly rate, %**	**Number of events**	**Yearly rate, %**	**Hazard ratio (95% CI)**	**p value**	**adjusted p value**
**Death**	3798	6.1	2270	3.9	1.673 (1.596-1.754)*	<0.0001	<0.0001
**ESKD**	200	0.4	143	0.3	1.553 (1.253-1.925)	<0.0001	<0.0001
**Ischemic stroke or thromboembolism**	359	0.8	241	0.5	1.712 (1.454-2.016)	<0.0001	<0.0001
**Acute MI**	352	0.7	217	0.4	1.827 (1.543-2.164)	<0.0001	<0.0001
**AF**	1495	3.3	1235	2.6	1.336 (1.239-1.441)	<0.0001	<0.0001
**VT/VF/Cardiac arrest**	1033	1.8	807	1.3	1.39 (1.267-1.524)	<0.0001	<0.0001
**MI/stroke/HF/death**	5949	8.6	3959	5.9	1.503 (1.453-1.554)*	<0.0001	<0.0001
**MI, ischemic stroke or HF**	3219	4.8	2240	3.2	1.576 (1.494-1.664)	<0.0001	<0.0001
**Incident HF**	2224	5.0	1739	3.9	1.285 (1.213-1.362)*	<0.0001	<0.0001
**Hosp. for HF**	1804	2.8	1291	1.9	1.51 (1.406-1.622)	<0.0001	<0.0001

* Risk ratio is presented instead of hazard ratio since the proportional hazard assumption was violated.

Table 3. Baseline characteristics of patients with no diabetes or with diabetes after propensity score matching.

	No diabetes, after propensity-score matching			Diabetes, after propensity-score matching		
	**hsCRP ≥ 2 mg/L (n = 52802)**	**hsCRP < 2 mg/L (n = 9430)**	**Std diff. (%)**	**hsCRP ≥ 2 mg/L (n = 9168)**	**hsCRP < 2 mg/L (n = 9168)**	**Std diff. (%)**
**Age at Index, n (%)**	74.6 +/- 12.2	73.4 +/- 11.8	10	74.0 +/- 11.0	73.5 +/- 10.3	4.7
**Men, n (%)**	4584 (50%)	4651 (50.7%)	1.5	1824 (52.2%)	1865 (53.4%)	2.3
**Systolic BP (mm Hg), mean±SD**	131.7 +/- 23.1	132.8 +/- 22.5	4.7	133.6 +/- 23.1	135.4 +/- 22.5	8.1
**Diastolic BP (mm Hg), mean±SD**	72.4 +/- 13.9	73.5 +/- 13.4	8.1	70.0 +/- 13.5	71.8 +/- 13.1	13.4
**Body mass index (kg/m2), mean±SD**	26.9 +/- 5.7	26.7 +/- 5.2	2.9	28.8 +/- 6.9	28.2 +/- 5.7	9.6
**White, n (%)**	3220 (35.1%)	3303 (36%)	1.9	1207 (34.5%)	1150 (32.9%)	3.5
**Black or African American, n (%)**	565 (6.2%)	535 (5.8%)	1.4	291 (8.3%)	280 (8%)	1.1
**Asian, n (%)**	1352 (14.7%)	1321 (14.4%)	1	148 (4.2%)	169 (4.8%)	2.9
**Hispanic or Latino, n (%)**	103 (1.1%)	105 (1.1%)	0.2	160 (4.6%)	151 (4.3%)	1.2
**Unknown race, n (%)**	3824 (41.7%)	3803 (41.5%)	0.5	1722 (49.3%)	1750 (50.1%)	1.6
Comorbid conditions						
**Hypertension, n (%)**	4652 (50.7%)	4706 (51.3%)	1.2	2430 (69.5%)	2408 (68.9%)	1.4
**Smoker, n (%)**	896 (9.8%)	888 (9.7%)	0.3	521 (14.9%)	465 (13.3%)	4.6
**Overweight or obesity, n (%)**	594 (6.5%)	593 (6.5%)	0	524 (15%)	495 (14.2%)	2.4
**Dyslipidaemia, n (%)**	3818 (41.6%)	3833 (41.8%)	0.3	2218 (63.5%)	2167 (62%)	3
**Alcohol related diagnoses, n (%)**	100 (1.1%)	87 (0.9%)	1.4	30 (0.9%)	25 (0.7%)	1.6
**Heart failure, n (%)**	1404 (15.3%)	1396 (15.2%)	0.2	877 (25.1%)	883 (25.3%)	0.4
**Coronary artery disease, n (%)**	5142 (56.1%)	5160 (56.3%)	0.4	2269 (64.9%)	2239 (64.1%)	1.8
**Myocardial infarction, n (%)**	598 (6.5%)	575 (6.3%)	1	308 (8.8%)	312 (8.9%)	0.4
**Dilated cardiomyopathy, n (%)**	78 (0.9%)	76 (0.8%)	0.2	49 (1.4%)	45 (1.3%)	1
**Ischemic stroke, n (%)**	2433 (26.5%)	2380 (26%)	1.3	891 (25.5%)	870 (24.9%)	1.4
**Intracranial hemorrhage, n (%)**	161 (1.8%)	141 (1.5%)	1.7	68 (1.9%)	78 (2.2%)	2
**Atrial fibrillation or flutter, n (%)**	1427 (15.6%)	1451 (15.8%)	0.7	591 (16.9%)	556 (15.9%)	2.7
**Kidney disease, n (%)**	2367 (25.8%)	2354 (25.7%)	0.3	1322 (37.8%)	1319 (37.7%)	0.2
**Lung disease, n (%)**	2660 (29%)	2679 (29.2%)	0.5	1239 (35.5%)	1226 (35.1%)	0.8
**COPD, n (%)**	616 (6.7%)	637 (6.9%)	0.9	292 (8.4%)	262 (7.5%)	3.2
**Sleep apnoea syndrome, n (%)**	489 (5.3%)	486 (5.3%)	0.1	392 (11.2%)	370 (10.6%)	2
**Peripheral vascular disease, n (%)**	600 (6.5%)	635 (6.9%)	1.5	389 (11.1%)	365 (10.4%)	2.2
**Previous cancer, n (%)**	1798 (19.6%)	1770 (19.3%)	0.8	833 (23.8%)	834 (23.9%)	0.1
**Anemia, n (%)**	1046 (11.4%)	985 (10.7%)	2.1	606 (17.3%)	579 (16.6%)	2.1
Biology measurements						
**Total cholesterol (mg/dL), mean±SD**	167.2 +/- 44.4	164.9 +/- 48.4	4.9	161.4 +/- 49.1	157.0 +/- 49.9	8.9

**LDL cholesterol (mg/dL), mean±SD**	91.6 +/- 36.3	89.0 +/- 37.0	7.3	86.4 +/- 37.5	83.3 +/- 38.2	8.2
**HDL cholesterol (mg/dL), mean±SD**	50.4 +/- 16.3	51.4 +/- 17.6	5.9	47.4 +/- 15.3	47.9 +/- 17.0	3.2
**Triglyceride (mg/dL), mean±SD**	127.0 +/- 78.8	123.5 +/- 88.7	4.2	146.8 +/- 119.5	143.5 +/- 99.2	3
**Hemoglobin A1c (%), mean±SD**	5.9 +/- 1.1	5.8 +/- 1.2	14.8	7.1 +/- 1.8	6.8 +/- 1.9	17.5
**Estimated GFR (MDRD, ml/min), mean±SD**	50.3 +/- 17.0	50.8 +/- 14.9	3.1	47.5 +/- 19.0	48.2 +/- 17.0	3.7
**Albuminuria (mg/g), mean±SD**	788.2 +/- 5257.2	1164.7 +/- 11302.6	4.3	780.9 +/- 3544.9	401.2 +/- 1033.2	14.5
**Albuminuria >=200 mg/g, n (%)**	53 (0.6%)	42 (0.5%)	1.7	143 (4.1%)	156 (4.5%)	1.8
**hsCRP (mg/L), mean±SD**	38.4 +/- 61.1	0.8 +/- 0.5	87	45.0 +/- 65.2	0.9 +/- 0.5	95.7
**Hemoglobin (g/dl), mean±SD**	12.6 +/- 2.1	13.1 +/- 2.0	19.9	12.2 +/- 2.1	12.7 +/- 2.1	20.2
Baseline treatments						
**Beta Blockers, n (%)**	3747 (40.9%)	3727 (40.7%)	0.4	1740 (49.8%)	1737 (49.7%)	0.2
**Calcium Channel Blockers, n (%)**	2925 (31.9%)	2871 (31.3%)	1.3	1427 (40.8%)	1372 (39.3%)	3.2
**ACE Inhibitors, n (%)**	1828 (19.9%)	1779 (19.4%)	1.3	846 (24.2%)	800 (22.9%)	3.1
**Angiotensin II Inhibitors, n (%)**	2079 (22.7%)	2056 (22.4%)	0.6	1063 (30.4%)	1060 (30.3%)	0.2
**Digitalis glycosides, n (%)**	242 (2.6%)	198 (2.2%)	3.1	88 (2.5%)	53 (1.5%)	7.1
**Diuretics, n (%)**	2808 (30.6%)	2757 (30.1%)	1.2	1238 (35.4%)	1232 (35.3%)	0.4
**Lipid lowering drugs, n (%)**	4473 (48.8%)	4448 (48.5%)	0.5	2076 (59.4%)	2051 (58.7%)	1.5
**Glucose-lowering therapy, n (%)**	1248 (13.6%)	1201 (13.1%)	1.5	2004 (57.3%)	2013 (57.6%)	0.5
**Insulin, n (%)**	952 (10.4%)	899 (9.8%)	1.9	1317 (37.7%)	1278 (36.6%)	2.3
**Non-insulin glucose-lowering therapy, n (%)**	520 (5.7%)	494 (5.4%)	1.2	1531 (43.8%)	1582 (45.3%)	2.9
**Metformin, n (%)**	327 (3.6%)	331 (3.6%)	0.2	1073 (30.7%)	1038 (29.7%)	2.2
**Sulfonylureas, n (%)**	141 (1.5%)	142 (1.5%)	0.1	628 (18%)	605 (17.3%)	1.7
**GLP-1 receptor agonists, n (%)**	39 (0.4%)	38 (0.4%)	0.2	166 (4.7%)	165 (4.7%)	0.1
**DPP4 inhibitors, n (%)**	134 (1.5%)	114 (1.2%)	1.9	598 (17.1%)	613 (17.5%)	1.1
**SGLT2 inhibitors, n (%)**	98 (1.1%)	94 (1%)	0.4	261 (7.5%)	257 (7.4%)	0.4
**Thiazolidinediones, n (%)**	19 (0.2%)	30 (0.3%)	2.3	137 (3.9%)	153 (4.4%)	2.3
**Antiplatelet therapy, n (%)**	4523 (49.3%)	4473 (48.8%)	1.1	1920 (54.9%)	1885 (53.9%)	2
**Anticoagulant, n (%)**	539 (5.9%)	537 (5.9%)	0.1	312 (8.9%)	287 (8.2%)	2.6

Table 4. Interaction analysis for clinical outcomes according to diabetes or no diabetes

		hsCRP ≥ 2 mg/L (n = 12663)			hsCRP < 2 mg/L (n = 12663)				
		Number of patients	Number of events	Yearly rate, %	Number of patients	Number of events	Yearly rate, %	Hazard ratio (95% CI)	p value for interaction
**Death**	**No DM**	9168	1948	5.73	9168	1069	3.36	1.973 (1.831-2.126)	
	**DM**	3495	797	6.11	3495	432	3.60	2.101 (1.868-2.362)	0.38
**ESKD**	**No DM**	9168	59	0.23	9168	42	0.13	1.516 (1.021-2.252)	
	**DM**	3495	68	0.64	3495	56	0.50	1.387 (0.973-1.976)	0.74
**Ischemic stroke or thromboembolism**	**No DM**	9168	176	0.72	9168	119	0.42	1.664 (1.319-2.100)	
	**DM**	3495	73	0.66	3495	54	0.43	1.554 (1.093-2.210)	0.75
**Acute MI**	**No DM**	9168	148	0.59	9168	93	0.32	1.745 (1.346-2.262)	
	**DM**	3495	63	0.59	3495	40	0.34	1.789 (1.203-2.660)	0.92
**AF**	**No DM**	9168	835	3.30	9168	643	2.41	1.397 (1.260-1.548)	
	**DM**	3495	321	3.28	3495	244	2.42	1.488 (1.259-1.758)	0.53
**VT/VF/Cardiac arrest**	**No DM**	9168	518	1.54	9168	376	1.14	1.46 (1.279-1.668)	
	**DM**	3495	221	1.86	3495	187	1.34	1.309 (1.077-1.590)	0.36
**MI/stroke/HF/death**	**No DM**	9168	3038	7.90	9168	1983	5.33	1.682 (1.590-1.780)	
	**DM**	3495	1218	8.14	3495	781	5.34	1.773 (1.621-1.940)	0.33
**MI, ischemic stroke or HF**	**No DM**	9168	1576	4.12	9168	1137	2.83	1.476 (1.368-1.593)	
	**DM**	3495	639	4.05	3495	498	3.10	1.398 (1.244-1.572)	0.45
**Incident HF**	**No DM**	9168	1189	4.53	9168	837	3.24	1.556 (1.425-1.700)	
	**DM**	3495	458	5.10	3495	409	4.71	1.232 (1.078-1.408)	0.004
**Hosp. for HF**	**No DM**	9168	759	2.10	9168	565	1.52	1.422 (1.275-1.586)	
	**DM**	3495	378	2.52	3495	311	2.05	1.318 (1.135-1.532)	0.42
